# Treatment of the mandibular shift in an adult woman and the diagnostic value of joint space index: a case report

**DOI:** 10.1186/s40001-020-00451-0

**Published:** 2020-10-22

**Authors:** Kai Xia, Wentian Sun, Liyuan Yu, Xinqi Huang, Zhihe Zhao, Jun Liu

**Affiliations:** 1 State Key Laboratory of Oral Diseases & National Clinical Research Center for Oral Diseases, West China Hospital of Stomatology, Sichuan University, No. 14, 3rd Section, South Renmin Road, Chengdu, 610041 Sichuan China; 2 Department of Orthodontics, West China Hospital of Stomatology, Sichuan University, No. 14, 3rd Section, South Renmin Road, Chengdu, 610041 Sichuan China

**Keywords:** Adult treatment, Asymmetry, Joint space index, Case report

## Abstract

**Background:**

Mandibular deviations are common clinical complaints. The orthodontic or orthognathic treatment of mandibular deviations is tricky because a comprehensive diagnosis, especially a functional one, is difficult to make. A inaccurate diagnosis may lead to a compromised and unstable treatment outcome.

**Case presentation:**

This article describes the diagnosis and treatment of a woman with a mandibular deviation and facial skeletal asymmetry. By eliminating the disharmony of the arch form with elastics and bite turbos, her esthetic and functional outcomes improved. Cone-beam CT (CBCT) and Joint Space Index (JSI) analyses served as the diagnostic approaches and outcome evaluation methods before and after treatment.

**Conclusions:**

A condyle position displacement could be an indication of functional deviation. JSI analysis is a quantitative and convenient choice to compare condyle relative positions.

## Background

Mandibular deviations are common malocclusions in orthodontic clinical practice characterized by facial asymmetry and chin and dental midline deviations. The reported prevalence of asymmetries in adults is 44.8% [1]. Mandibular deviations are one of the essential factors affecting facial attractiveness, especially in terms of lateral displacement of the chin [1, 2]. Interventions with orthodontic or orthognathic approaches should be carefully chosen according to the extent and the etiology of deviations [3–5]. The etiology factors are generally classified as dental, skeletal, and functional [6]. Comprehensive diagnoses are difficult to make and only become trickier for functional diagnoses. If the diagnosis is not accurate, the treatment outcome will be compromised and unstable [4, 7]. This case report describes the diagnosis and treatment of a woman with a functional mandible deviation and facial asymmetry. Using cone-beam CT (CBCT) and a Joint Space Index (JSI) analysis, we arrived at a reliable diagnosis to guide the choice of treatment. Treatment with elastics and bite turbos finally resolved her deviation by eliminating the disharmony of the arch form and relocation of the condyles. The treatment improved her esthetics and function, what is more, a promising diagnostic method for functional deviations deserves more attention.

## Case presentation

### Diagnosis and etiology

**Fig. 1:**
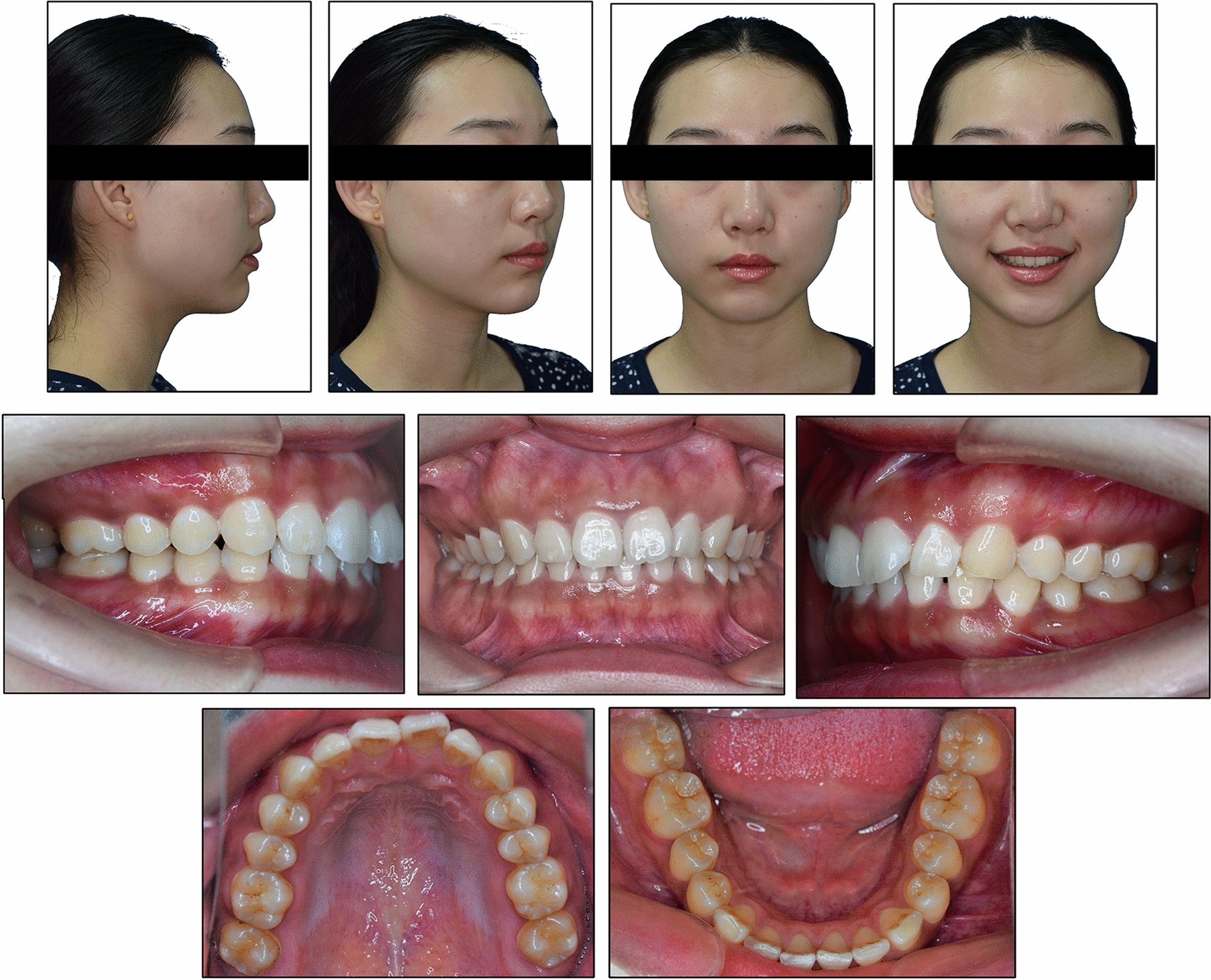
Pretreatment facial and intraoral photographs

**Fig. 2:**
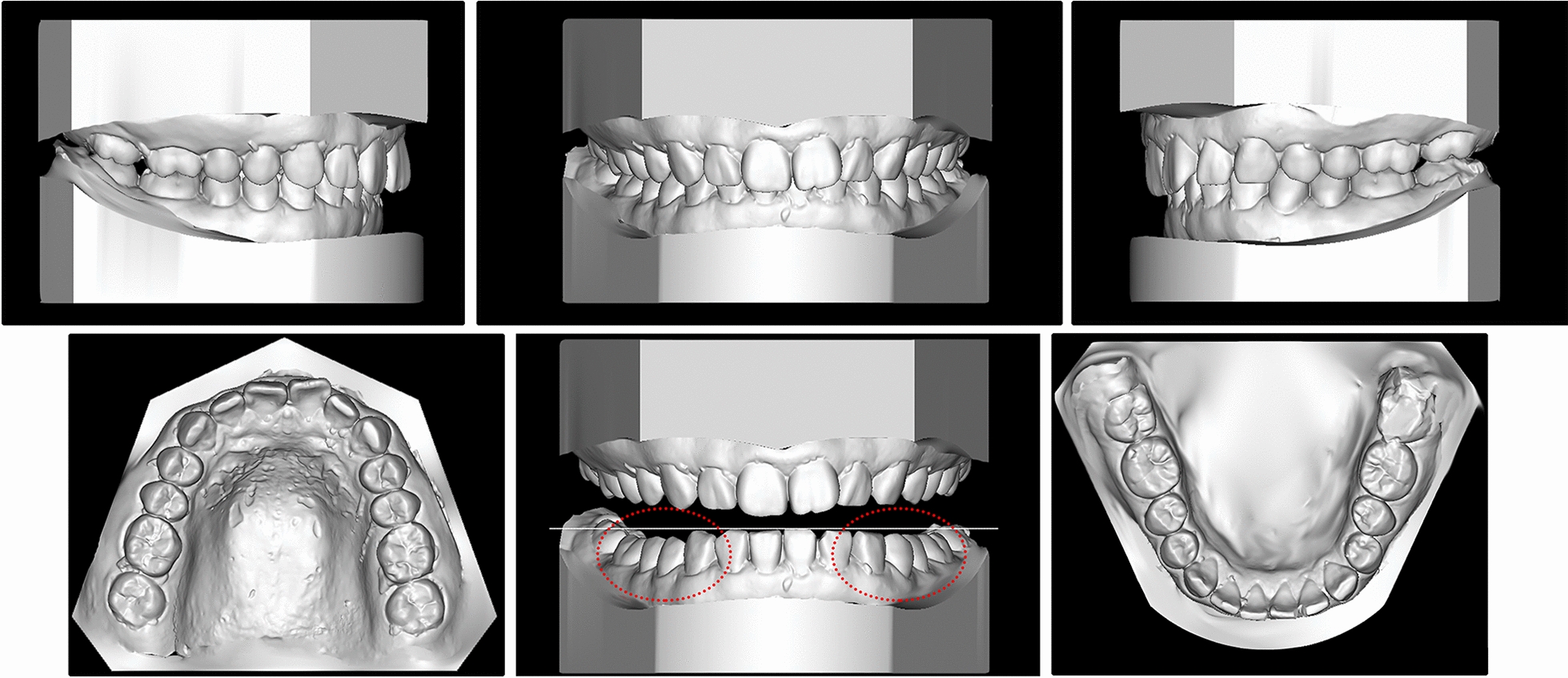
Pretreatment dental casts

**Fig. 3:**
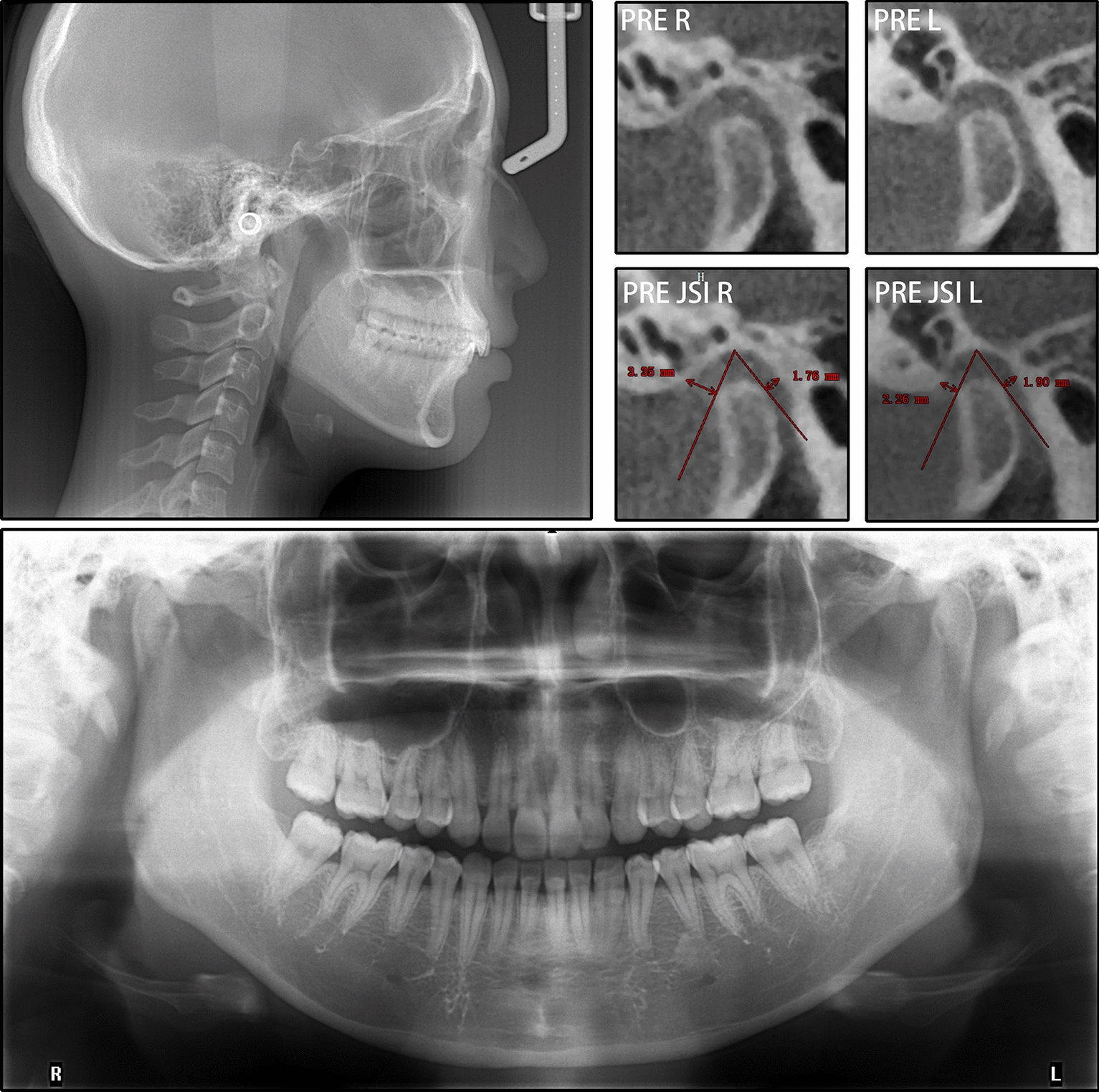
Pretreatment radiographs and bilateral TMJ JSIs

**Fig. 4:**
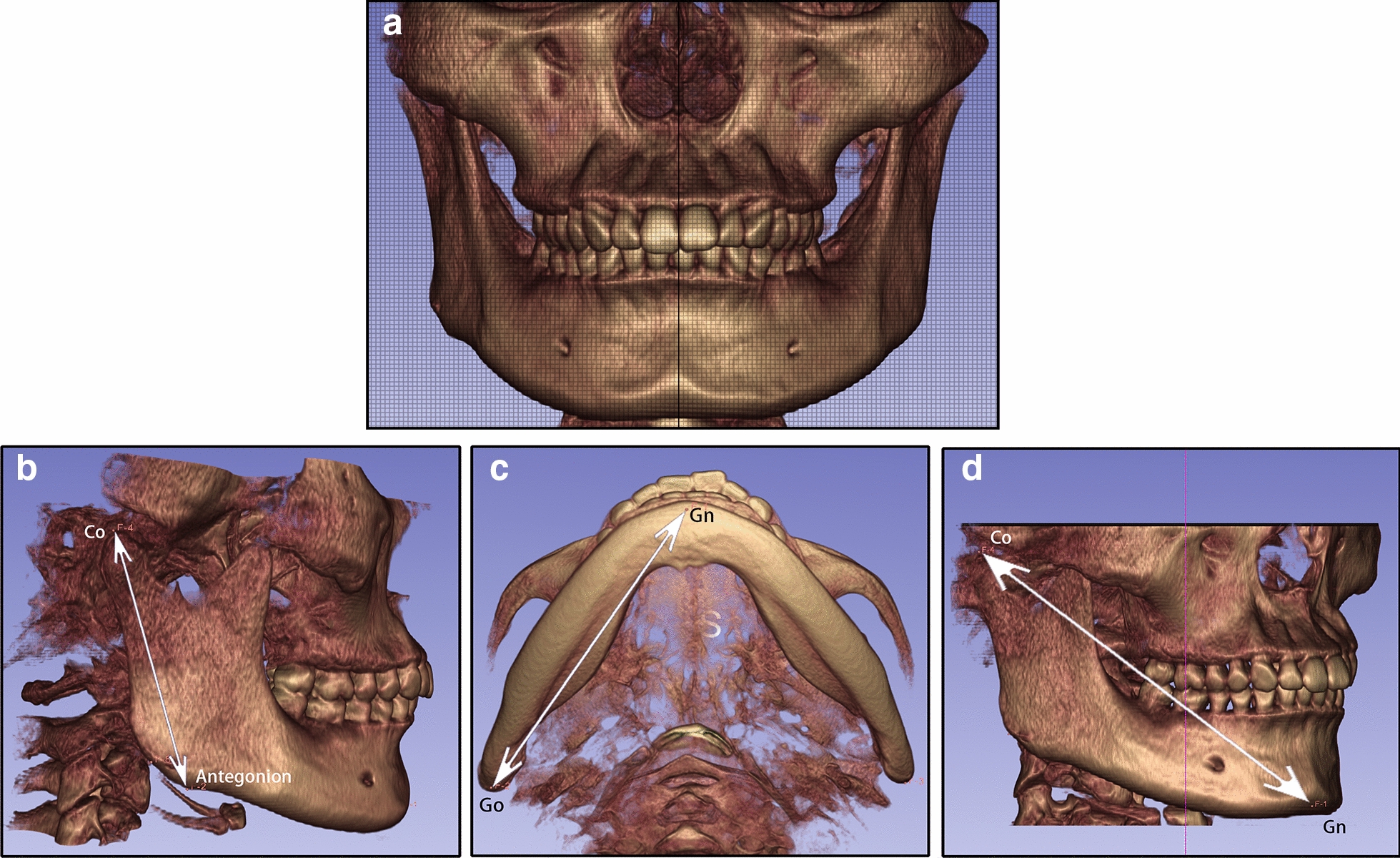
Three-dimensional reconstructions and measurements of the mandible. **a** Orientated three-dimensional reconstructions of the craniomaxillofacial complex (The grid has a width of 1 mm) showed that the mandible deviated 1.5 mm to the right. **b** The ramus length, **d** the body length and **d** the total length of the mandible

The patient was 23-year-old healthy woman with chief complaints of a mandibular deviation and crowded dentition. The facial evaluation showed an asymmetric face with a mandible shift to the right side and a soft-tissue asymmetry. A convex profile and a deep mentolabial sulcus were noted. The maxillary dental midline was coincident with the facial midline; whereas, the mandibular dental midline deviated 1.5 mm to the right. Intraorally, a half-step Class II molar and canine relationship was observed on the right; a Class I molar and canine relationship was observed on the left. The dentition was mildly crowded in both arches with a 5-mm overbite and a 4-mm overjet (Fig. 1). The dental cast analysis showed a normal Bolton index but a disharmonious arch width between the maxillary and mandibular arch: the mandibular arch width was 1.5 mm wider than the maxillary arch in the premolar region. A reduced vertical height of the posterior region on the right side was observed (Fig. 2). The cephalometric analysis showed a normal vertical skeletal pattern (Frankfort-mandibular plane angle (FMA), 28.2°; sella-nasion to Gonion-Gnathion angle (SN-GoGn), 33.5°) and a mild Class II sagittal pattern (sella-nasion A-point angle (SNA), 82.9; sella-nasion B-point angle (SNB), 79.0; A-point-nasion-B-point angle (ANB), 3.9). The incisors were slightly retruded in the maxillary and mandibular regions (interincisal angle, 134.6°; 102.3° from the maxillary incisor to SN; incisor mandibular plane angle (IMPA), 89.6°). The JSI was calculated with CBCT as previously described [8]. The JSI of the right condyle was smaller than that of the left (right, − 30.7, left, − 8.7), which meant that the right condyle was in a more posterior position than the left condyle (Fig. 3; Table 1, 2). The three-dimensional reconstruction of the CBCT image showed that a right-deviated mandible (skeletal landmarks sella, basion, and anterior nasal spine were used as midsagittal plane reference to orient CBCT images and measure asymmetry [9]), the ramus, body, and total length of the right side were mildly shorter than those of the left side (Fig. 4; Table 3). That is, her deviation presented both functional and skeletal factors.

**Table 1:** Cephalometric measurements

Measurement	Normal	Before treatment	After treatment
SNA (°)	81.7 ± 2.5	82.9	82.5
SNB (°)	78.9 ± 2.2	79.0	78.9
ANB (°)	2.8 ± 1.2	3.9	3.6
SN-MP (°)	32.9 ± 4.2	33.5	34.5
FMA (°)	29.0 ± 3.5	28.2	29.3
Y-axis (°)	63.5 ± 3.2	64.1	64.9
S-Go/N-Me	65.9 ± 3.8	63.3	62.8
ANS-Me/N-Me	53.3 ± 1.8	57.5	57.7
U1-L1 (°)	123.2 ± 6.2	134.6	129.5
U1-SN (°)	105.1 ± 6.2	102.3	103.7
IMPA (°)	95.4 ± 4.7	89.6	92.3
UL-EP (mm)	− 0.5 ± 1.9	− 1.9	− 2.0
LL-EP (mm)	1.3 ± 1.9	1.3	1.53
Z angle (°)	74.1 ± 4.6	68.3	69.5

*S* sella, *N* nasion, *A* A-point, *B* B-point, *MP* mandibular plane, *SN* sella-nasion plane, *FMA* Frankfort-mandibular plane angle, *IMPA* incisor mandibular plane angle, *EP* esthetic plane, *UL* upper lip, *LL* lower lip

**Table 2:** Bilateral joint space measurements

	Right TMJ			Left TMJ		
	Anterior JS (mm)	Posterior JS (mm)	JSI	Anterior JS (mm)	Posterior JS (mm)	JSI
Pretreatment	3.35	1.76	− 30.7	2.26	1.90	− 8.7
Posttreatment	2.59	2.96	6.7	1.47	2.44	19.4
Relative change (Post–pre)	− 0.76	1.20	37.4	-0.79	0.54	28.1

*TMJ* temporomandibular joint, *JS* joint space, *JSI* Joint Space Index

**Table 3:** Measurements of the mandible

	Ramus length (mm)	Body length (mm)	Total length (mm)
Right mandible	65.5	90.8	124.6
Left mandible	66.2	91.6	125.6
Relative difference (left–right)	0.7	0.8	1.0

### Treatment objectives

With a focus on the patient’s chief complaints, the primary objectives of treatment were to coordinate the dental midlines and correct the asymmetrical facial appearance. The other treatment objectives were to resolve the dental crowding, establish normal overbite and overjet relationships, and obtain an ideal occlusion.

### Treatment alternatives

The patient was told that if orthodontic treatment could not correct her asymmetric appearance, surgery could be an alternative choice. However, because of the surgical risks and costs of surgical intervention, surgical treatment was not recommended for this patient.

### Treatment progress

**Fig. 5:**
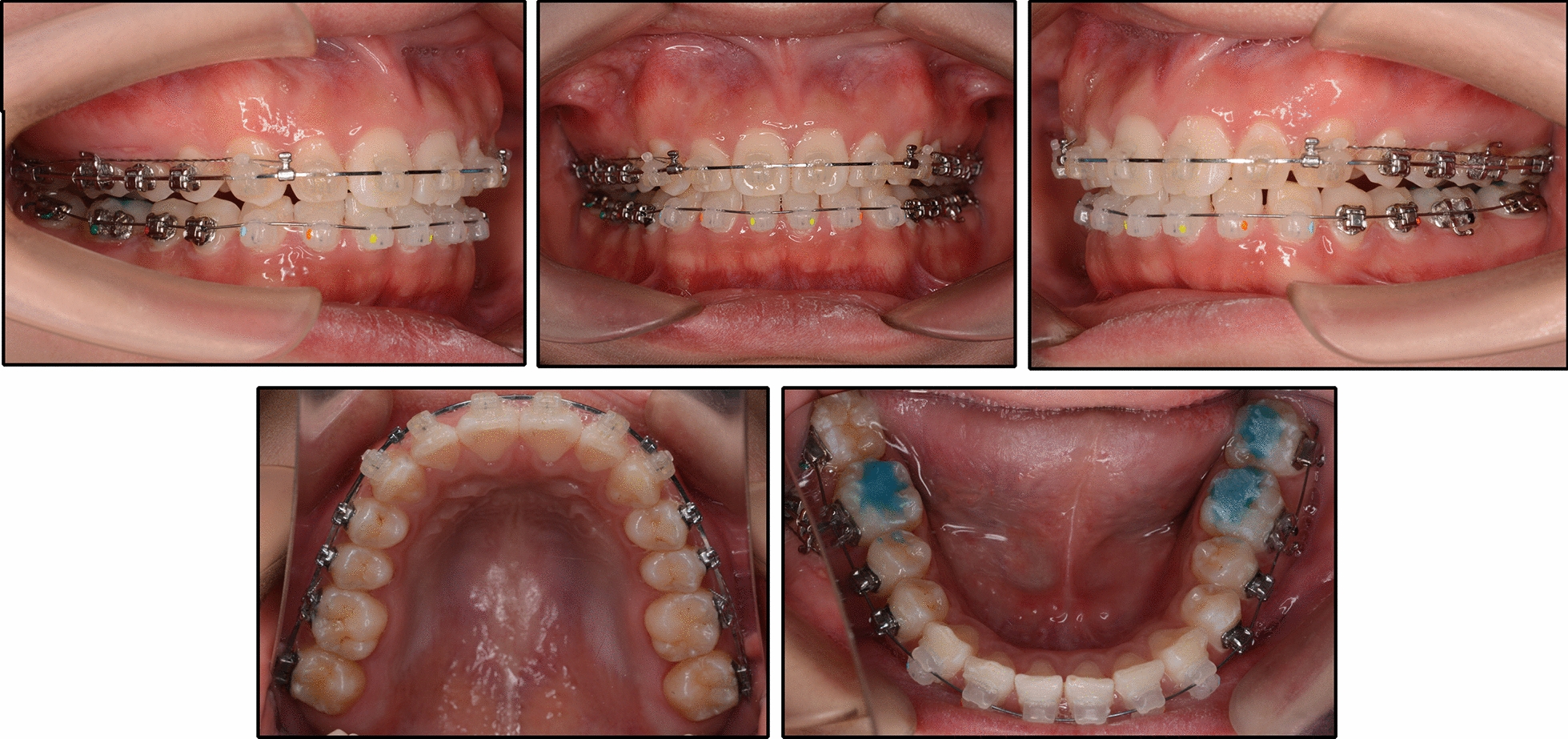
Intraoral photographs during treatment: Bite turbos were added to control the vertical height of the lower molars

**Fig. 6:**
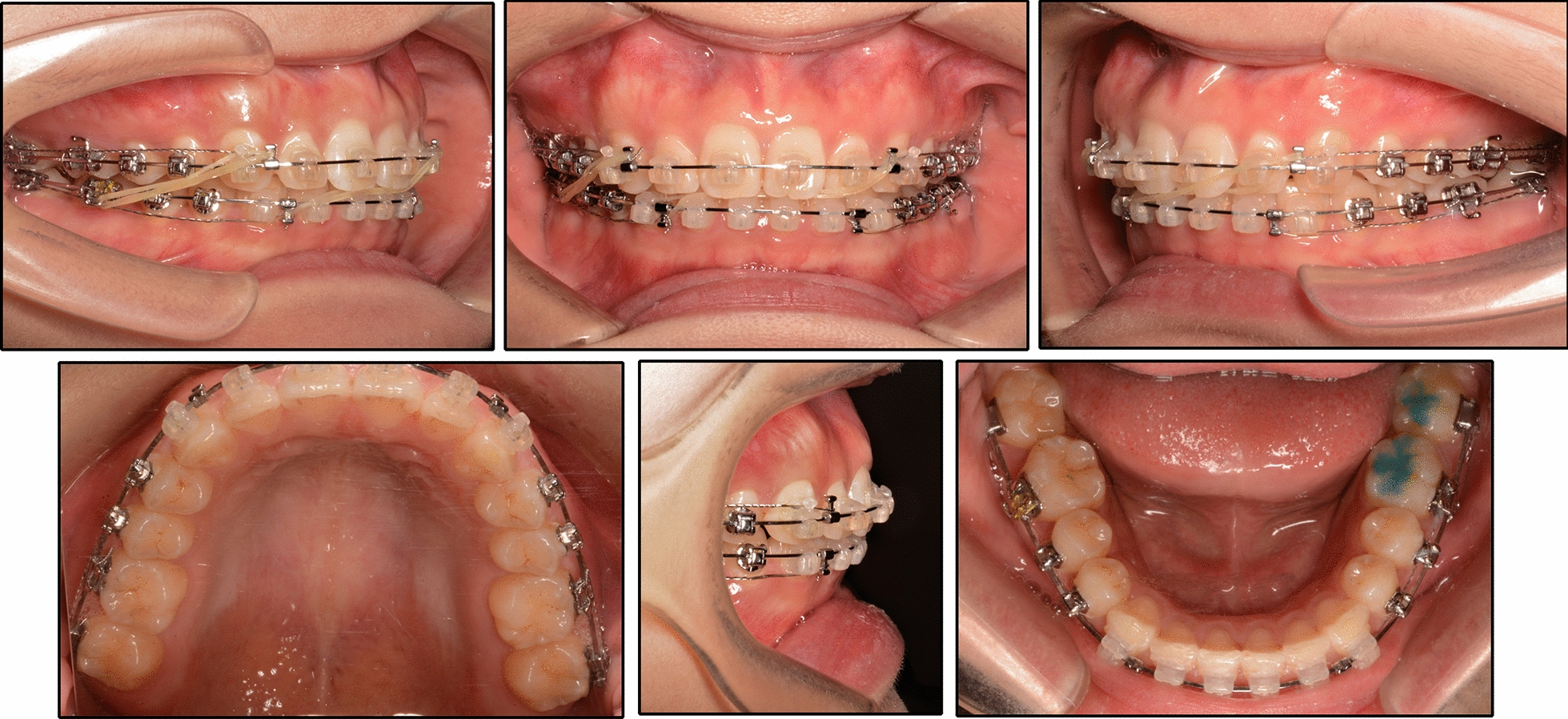
Intraoral photographs during treatment: A unilateral Class II elastic and an anterior diagonal elastic were used to correct the mandibular functional shift

To meet her esthetic demand, a ceramic preadjusted bracket (0.022-inch slot, Damon Clear; Ormco, Orange, CA) was placed on the labial side of both arches. A 0.014-inch round nickel–titanium wire was used to initiate alignment. At 7 months, a 0.018 × 0.025-inch stainless steel working wire and a 0.014-inch round nickel–titanium wire were placed in the upper and lower arch, respectively. Bite turbos were added to the occlusal surfaces of the mandibular right first molar and left molars to control the vertical height of the lower molars (Fig. 5). After 5 months, both arches were leveled and aligned, and the arch forms were coordinated by matching the archwires with archform chart. A unilateral Class II elastic (Penguin, 5/16-in, 3.5-oz; Ormco) and an anterior diagonal elastic (Penguin, 5/16-in, 3.5-oz; Ormco) were used from the upper right side to the lower left side to correct the mandibular functional shift (Fig. 6). Furthermore, the bite turbos on the mandibular right first molar were progressively reduced to reestablish a normal vertical dimension and ideal bilateral occlusion. After 8 months, the dental midlines were coordinated with Class I molar and canine relationships, and the use of the elastics was stopped. Short-term efficacy stability was observed for another 3 months before removing the fixed appliances. The treatment period was 24 months, and a Hawley retainer was used for retention.

### Treatment results

**Fig. 7:**
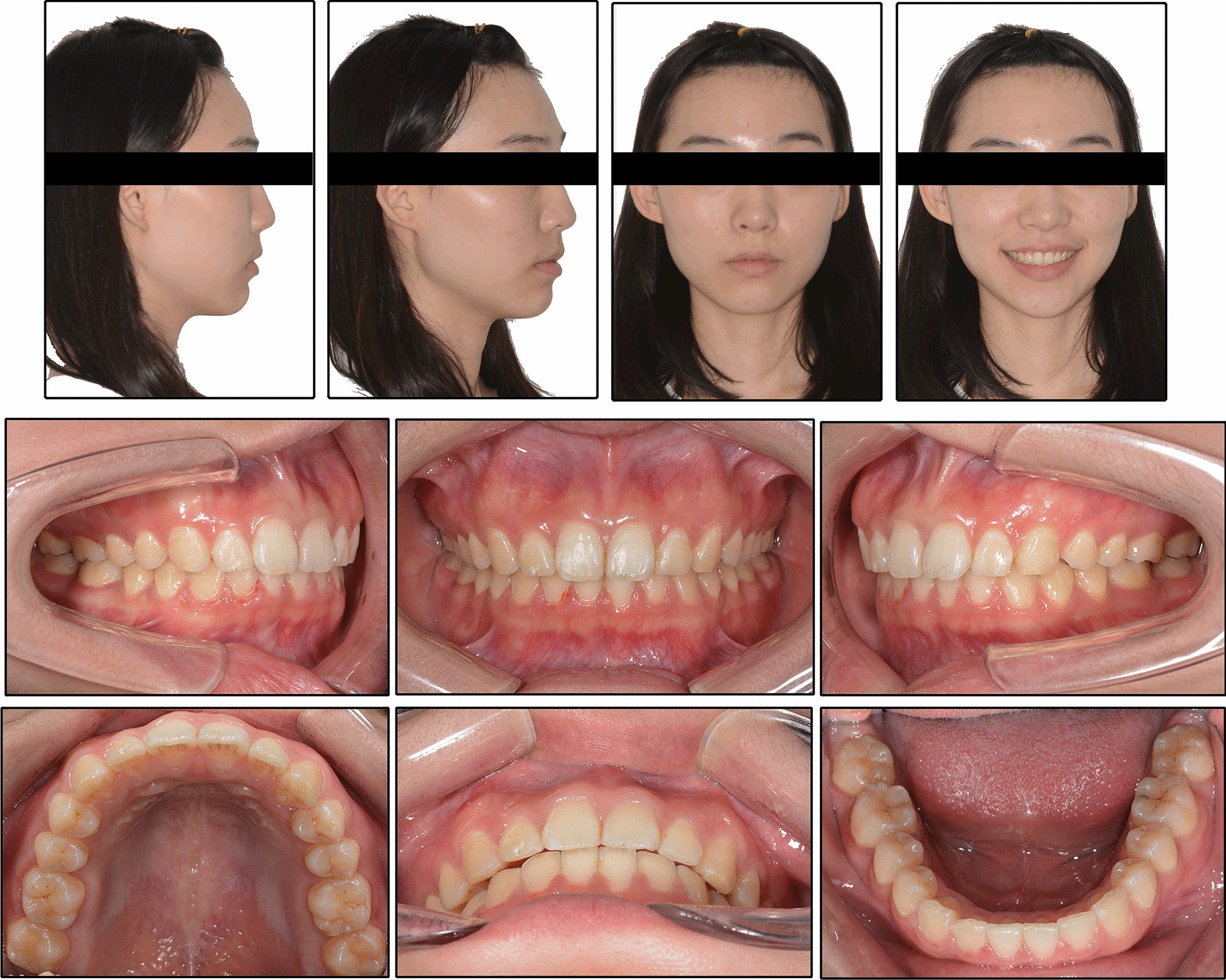
Post-treatment facial and intraoral photographs

**Fig. 8:**
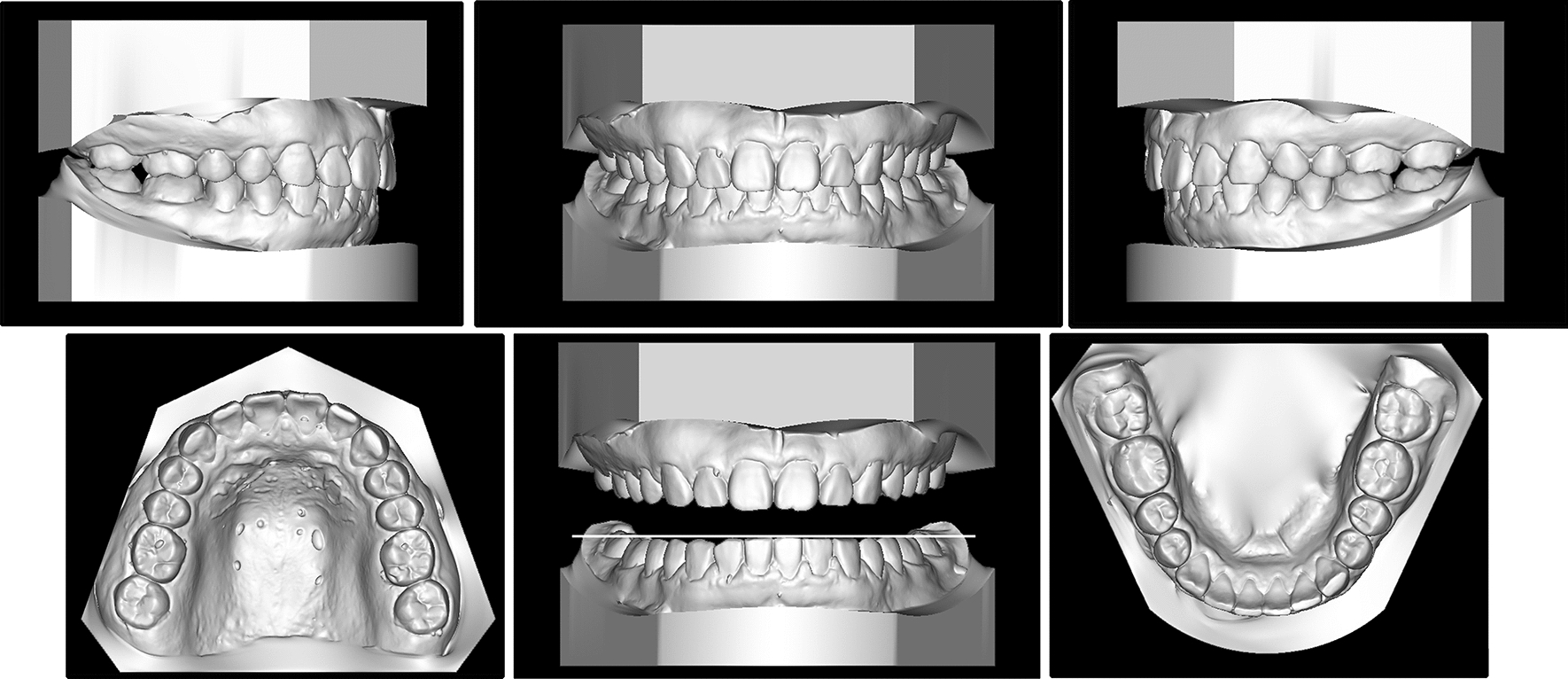
Post-treatment dental casts

**Fig. 9:**
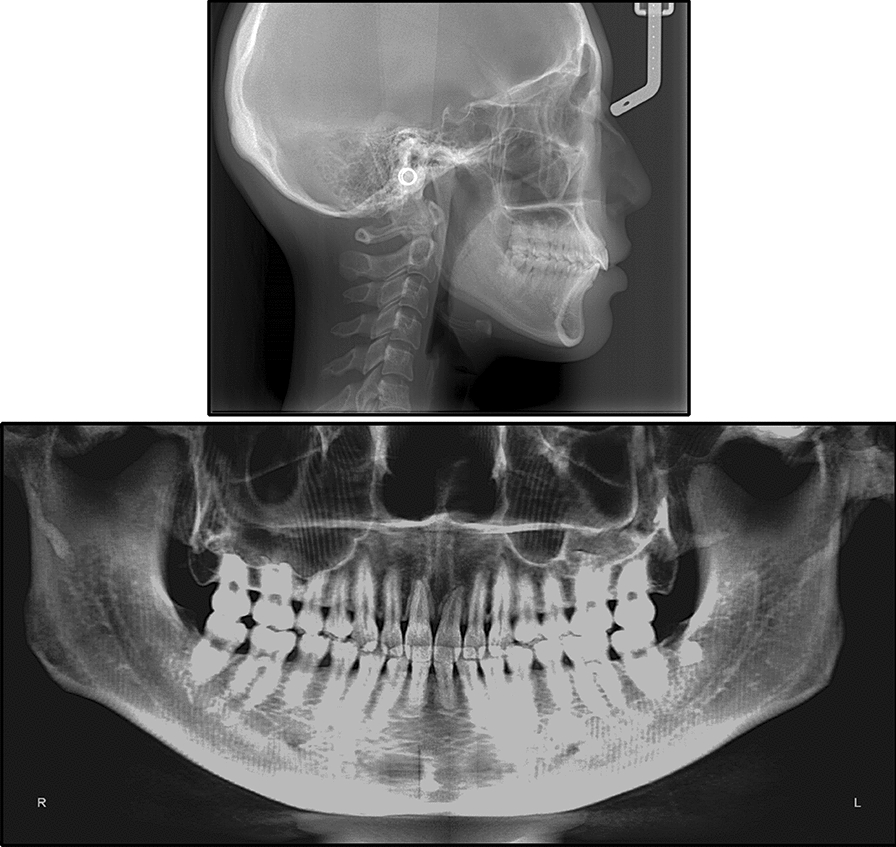
Post-treatment radiographs

**Fig. 10:**
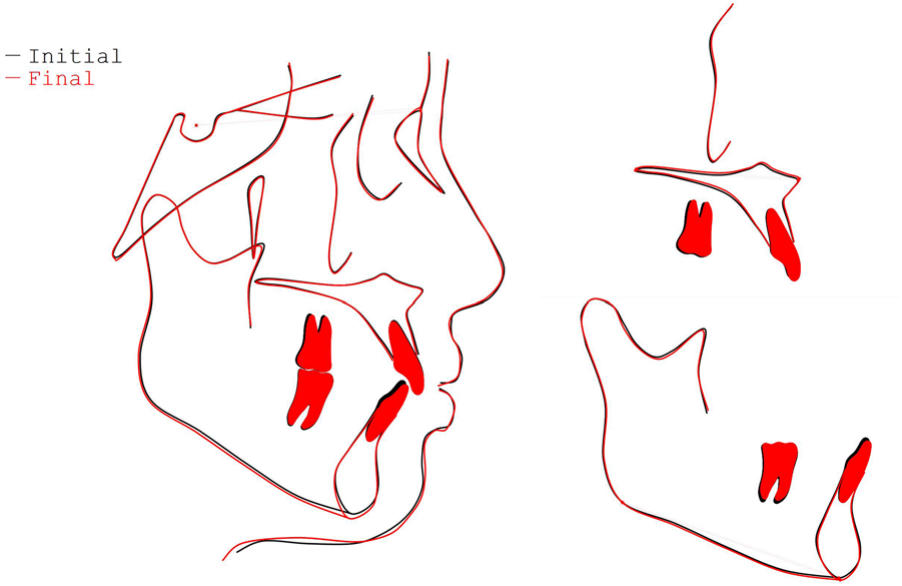
Superimpositions of the pretreatment (black) and post-treatment (red) lateral cephalometric tracings

**Fig. 11:**
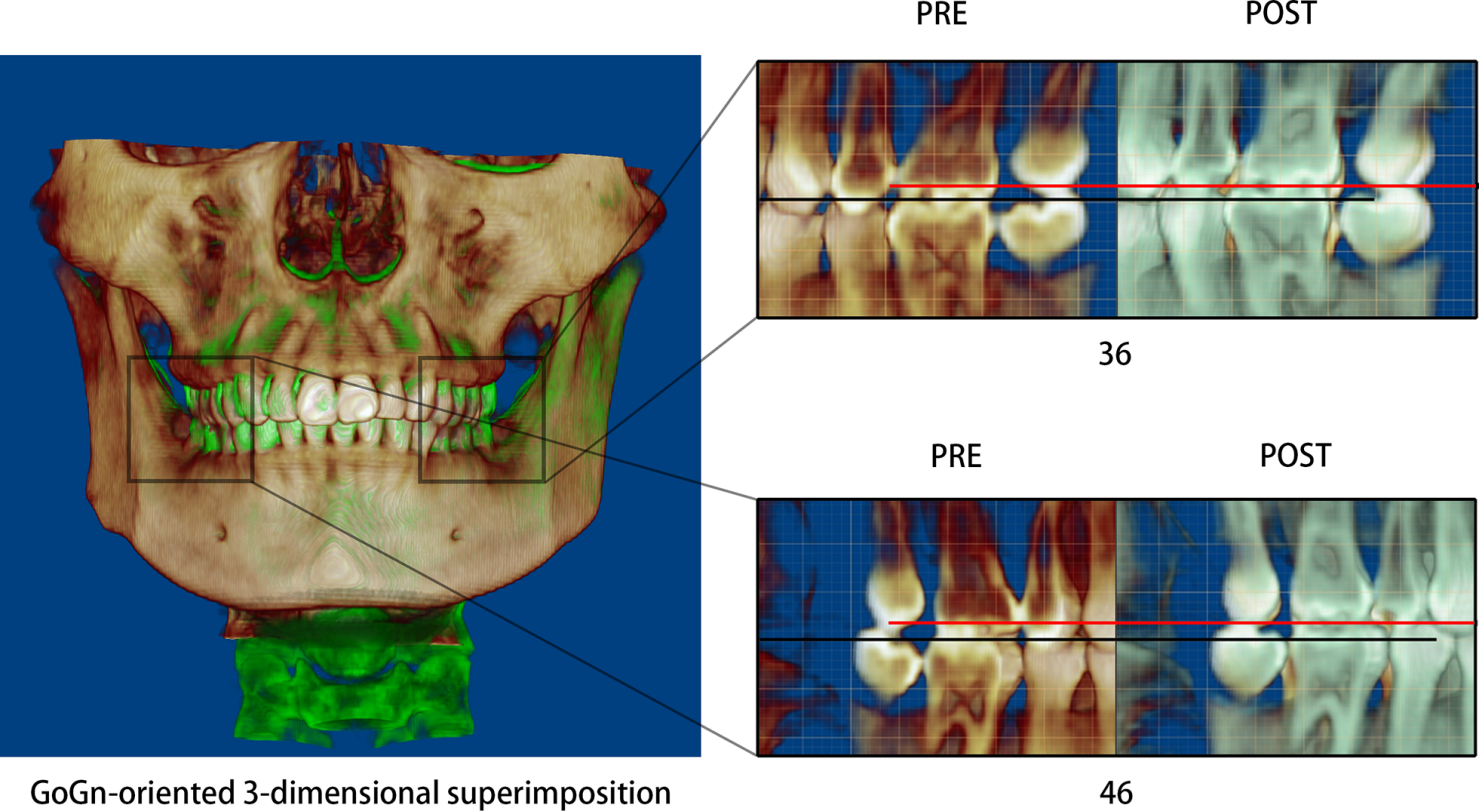
GoGn-oriented three-dimensional superimposition of the mandible reconstruction model indicating the post-treatment (red) molar extrusion compared with the pretreatment (black)

**Fig. 12:**
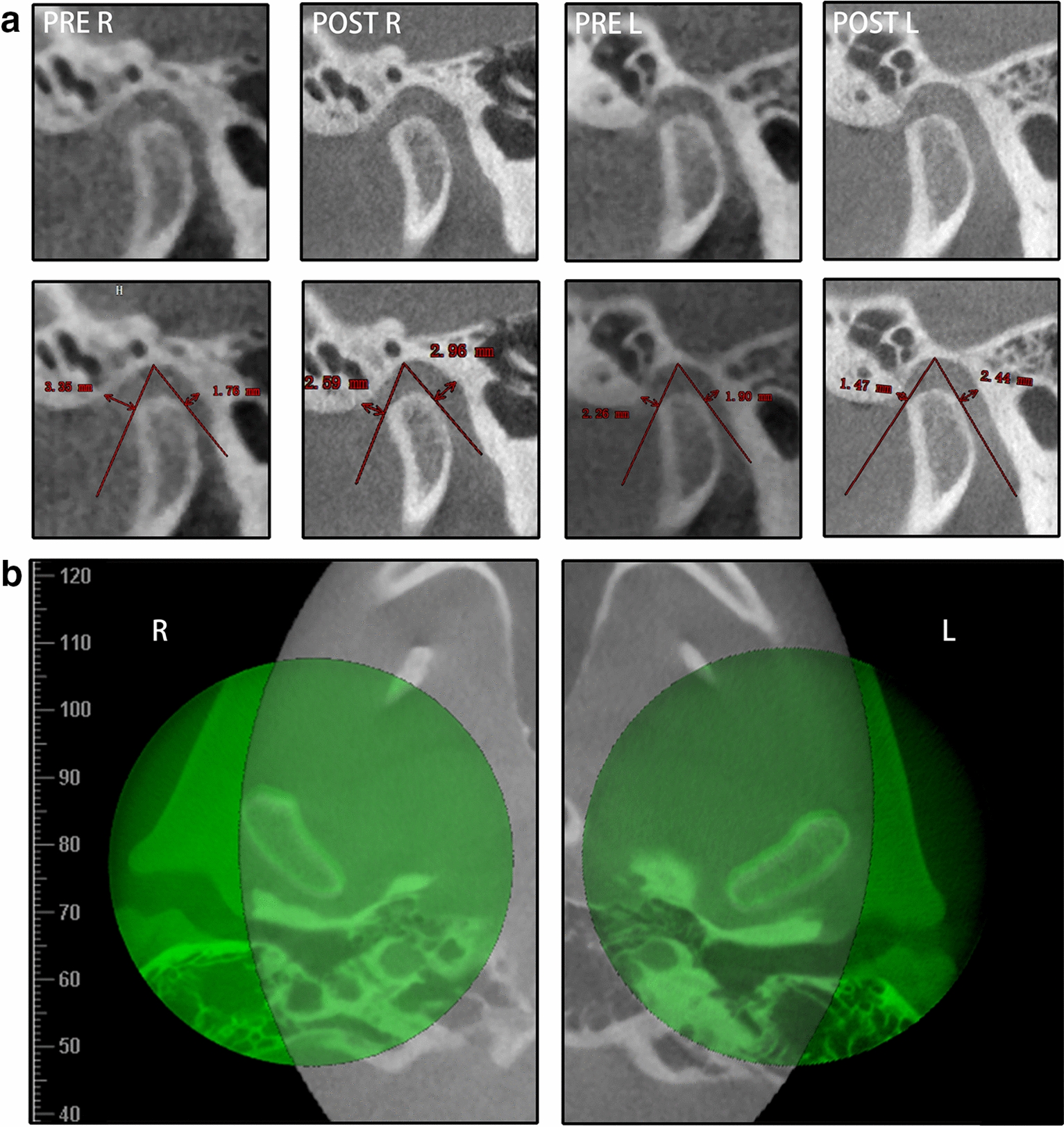
**a** Comparison of the pretreatment and post-treatment bilateral TMJ JSIs. **b** CBCT superimposition of the pretreatment (gray) and post-treatment (green) bilateral TMJs

**Fig. 13:**
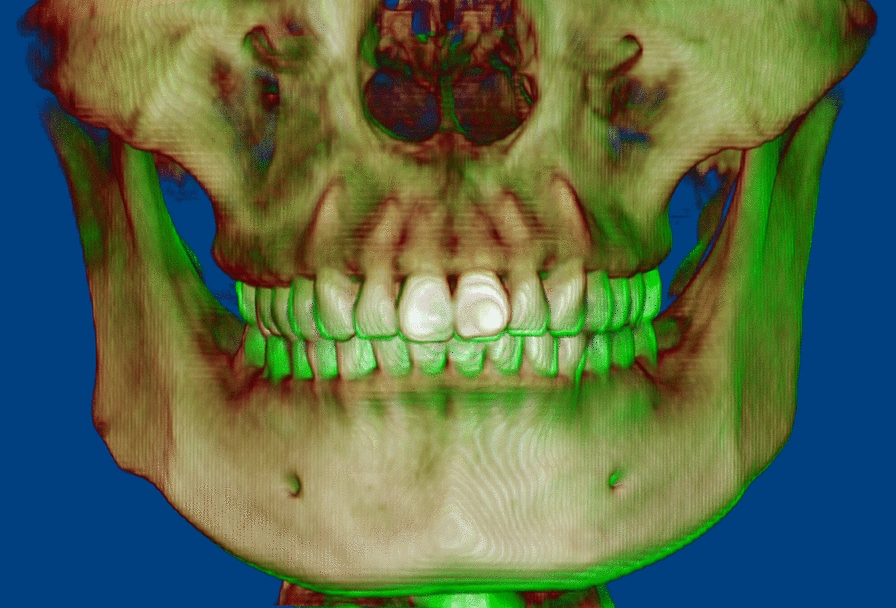
The three-dimensional superimposition based on cranial base and zygoma showed that the post-treatment (green) mandible is repositioned back to the left compared with the pretreatment (beige)

**Fig. 14:**
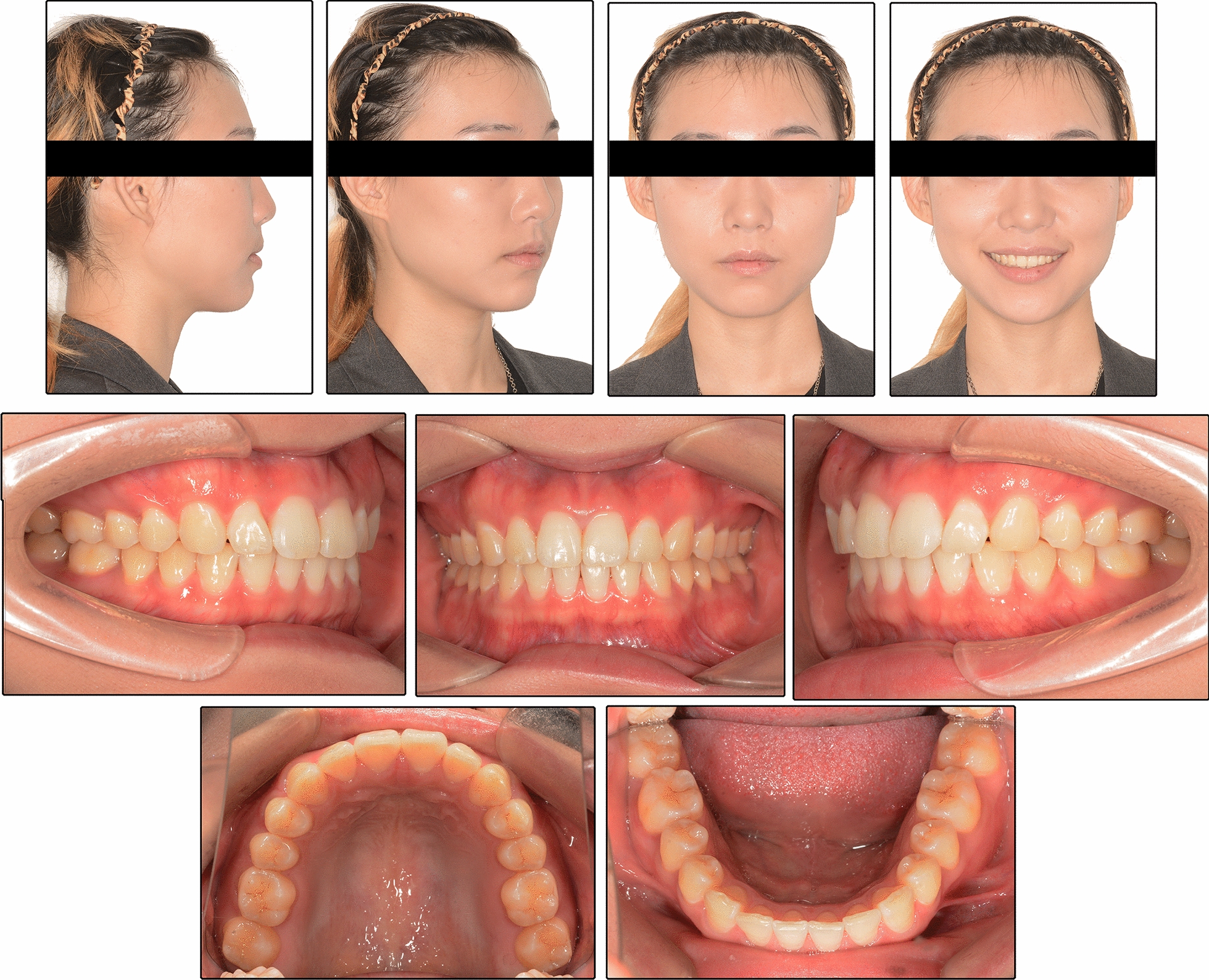
Facial and intraoral photographs after 14 months of retention

Class I molar and canine relationships with coincident dental and facial midlines and adequate overbite and overjet relationships were achieved. The facial photographs showed a symmetrical appearance (Fig. 7). The dental cast analysis indicated that the maxillary interpremolar (14–24) and intermolar (16–26) widths increased by 2.3 mm and 1.3 mm, respectively, and that the arch forms were coordinated between the two arches. The reduced vertical height of the right posterior region was also corrected (Fig. 8). No resorption or deformity in the condylar structure was found with CBCT of the temporomandibular joint (TMJ) regions. The panoramic radiographs showed parallel roots without root resorption (Fig. 9). The superimposition of the cephalometric radiographs showed normalization of the incisor inclination. A slight clockwise rotation of the mandibular plane was observed in response to the molar extrusions. A GoGn-oriented superimposition of three-dimensional mandible reconstructions also verified the extrusion. The right lower first molar and left lower first molar extruded 1.8 mm and 1.1 mm, respectively (Figs. 10 and 11). The JSI of both sides increased after treatment (Δright JSI = 37.4, Δleft JSI = 28.1), indicating that the right condyle presented a more significant anterior relocation than the left condyle (Fig. 12; Table 2). A three-dimensional superimposition based on cranial base and zygoma showed that asymmetry has improved as the mandible is repositioned back to the left (Fig. 13). The patient was satisfied with the result of the treatment, and did not complain of temporomandibular disorder (TMD) symptoms throughout the treatment period. After 14 months of retention, the patient came for a follow-up. Her symmetrical appearance and occlusion were still stable (Fig. 14).

## Discussion

Dental and facial asymmetries are common chief complaints in orthodontic patients. Class II subdivision malocclusions tend to lead to mandibular midlines not coincident with facial midlines [10]. The causes of this deviation are varied. Cassidy et al. found that most Class II subdivision patients exhibited some degree of mandibular skeletal asymmetry [10]. Li et al. investigated the origin of subdivision asymmetries with CBCT and dental cast analyses and found that functional factors such as a disharmonious arch width between the maxillary and mandibular dental arches in the premolar section accounted for the majority of the cases [11].

In the diagnosis process, we found that this patient had both skeletal and functional factors by means of JSI analysis and three-dimensional reconstructions, and we speculated that the long-term functional deviation might be the reason for her skeletal deviation, as previous studies indicated [12–15]. Functional deviations are usually examined by observing the coincidence of the dental midlines during mouth closure in the maximum intercuspal position, at initial contact, and at the mandibular rest position. Patients with functional deviations will show midline deviations between the initial contact and mouth closure. As this mild deviation is difficult to detect with direct observations, the condylar position indicator and mandibular monitoring could help to diagnose abnormal condylar position and mandibular movements [16–18]. However, the dentofacial complex shows exceptional adaptability to functional demands. A previous study indicated that this ability to adapt to functional deviations of the mandible leads to a functional rebalance [19, 20]. That is, long-term mandibular functional shifts and the associated mechanically forced displacement of the mandible in the closure path may be compensated by neuromuscular adaption. In clinical manifestations, the mandible directly moves into the intercuspal position (ICP) without interference from the teeth, and a mandibular midline deviation exists, regardless of the jaw position.

As different types of malocclusion have varying degrees of condyle displacement [21, 22], we predicted that the long-term mandibular functional shift also led to condyle displacement and that the condyle finally relocated because of a neuromuscular adaption. Since the centric relation (CR) theory was put forth, most of the controversies were related to the position of the condyle in the glenoid fossa, which is clinically invisible [23]. What is more, there is no established “gold standard” method of CR registration with high repeatability and operability [24]. To directly assess the condylar position, radiological evaluation is a preferable manner [25–27]. Magnetic resonance images (MRI) can be used to evaluate the position of the condyle and disk in the fossa, but it can not be commonly performed in oral clinical practice due to its accessibility and cost problems. With the advent of CBCT, high-definition and sensitive measurements of the condyle position became possible [26, 28, 29]. The combination of CBCT and JSI analysis enables a quantitative and convenient comparison of the relative condyle positions [30]. Research suggested that the physiologic JSI range for the condylar position is − 32.5 to 21.1 [31]. In this patient, the condyle on the shifted side (right) was in a posterior position on her first visit (JSI: right, − 30.7, and left, − 8.7). After the treatment, the right condyle was located in a relatively anterior position, and the mandible shift was also corrected (JSI: right, 6.7, and left, 19.4). Both condyles relocated anteriorly to the central position of the glenoid fossa, which is considered a relatively physiologic position [32].

Park et al. reported that mandible skeletal asymmetry presents dental compensation, such as vertical movement of the molars and a transverse cant of the occlusal plane [4]. Ishizaki et al. indicated that a reduced vertical height of the dentition on one side induces a mandibular lateral shift in a three-dimensional rotational manner [33]. Dental decompensation is an important part of mandible deviation treatment; otherwise, the effect will be compromised. With the use of bite turbo and unilateral Class II elastics in this patient, the reduced vertical height of the right posterior region was corrected. The bite turbo in the posterior area served two purposes: (1) to avoid the cusp-to-cusp contact and consequent unstable jaw position, and (2) to control the vertical height of the molar region with masticatory forces on the left side. In addition, a gradual reduction of the bite turbo thickness on the right side in combination with the elastics led to the vertical height recovery.

The molar was slightly extruded as a consequence of the reverse-curve arch wire. The sella-nasion mandibular plane angle (SN-MP) consequently increased by 1° due to the clockwise mandibular rotation. Interestingly, this rotation did not worsen the skeletal Class II relationships as the ANB remained unchanged. The repositioning of the condyle might compensate for this trend.

The newly acquired condyle and jaw position was retained with intercuspation occlusion. The skeletal facial asymmetry was partly compensated for by the recovery of the mandibular functional shift, as previously reported [34]. The treatment outcome was favorable after 14 months of retention. For functional deviation patients, wearing a Hawley retainer during the day for the first year and tooth positioner at night for 2–3 years could be an effective approach to stabilize the jaw position for retention.

In conclusion, the typical clinical manifestations of functional deviations are not easily observed, as they might be compensated by neuromuscular adaption. In this case, a condyle position displacement could be an indication of functional deviation. JSI analysis is a quantitative and convenient choice to compare condyle relative positions. Furthermore, the recovery of the vertical height in the molar region should be considered as an important part in the mandibular functional deviation treatment process.

## Data Availability

The data that support the findings of this study are available from the corresponding author upon reasonable request.

## Abbreviations

CBCT: Cone-beam computed tomography
JSI: Joint space index
ICP: Intercuspal position
CR: Centric relation
