# Correction to: Treatment of the mandibular shift in an adult woman and the diagnostic value of joint space index: a case report

**DOI:** 10.1186/s40001-020-00457-8

**Published:** 2020-11-12

**Authors:** Kai Xia, Wentian Sun, Liyuan Yu, Xinqi Huang, Zhihe Zhao, Jun Liu

**Affiliations:** 1 State Key Laboratory of Oral Diseases & National Clinical Research Center for Oral Diseases, West China Hospital of Stomatology, Sichuan University, No. 14, 3rd Section, South Renmin Road, Chengdu, 610041 Sichuan China; 2 Department of Orthodontics, West China Hospital of Stomatology, Sichuan University, No. 14, 3rd Section, South Renmin Road, Chengdu, 610041 Sichuan China

## Correction to: Eur J Med Res (2020) 25:50 https://doi.org/10.1186/s40001-020-00451-0

In the original version of the article, the pretreatment TMJ images demonstrated in Fig. 12a had been shown in Fig. 3. Therefore, the pretreatment TMJ images were deleted in Fig. 12a.

**Fig. 12:**
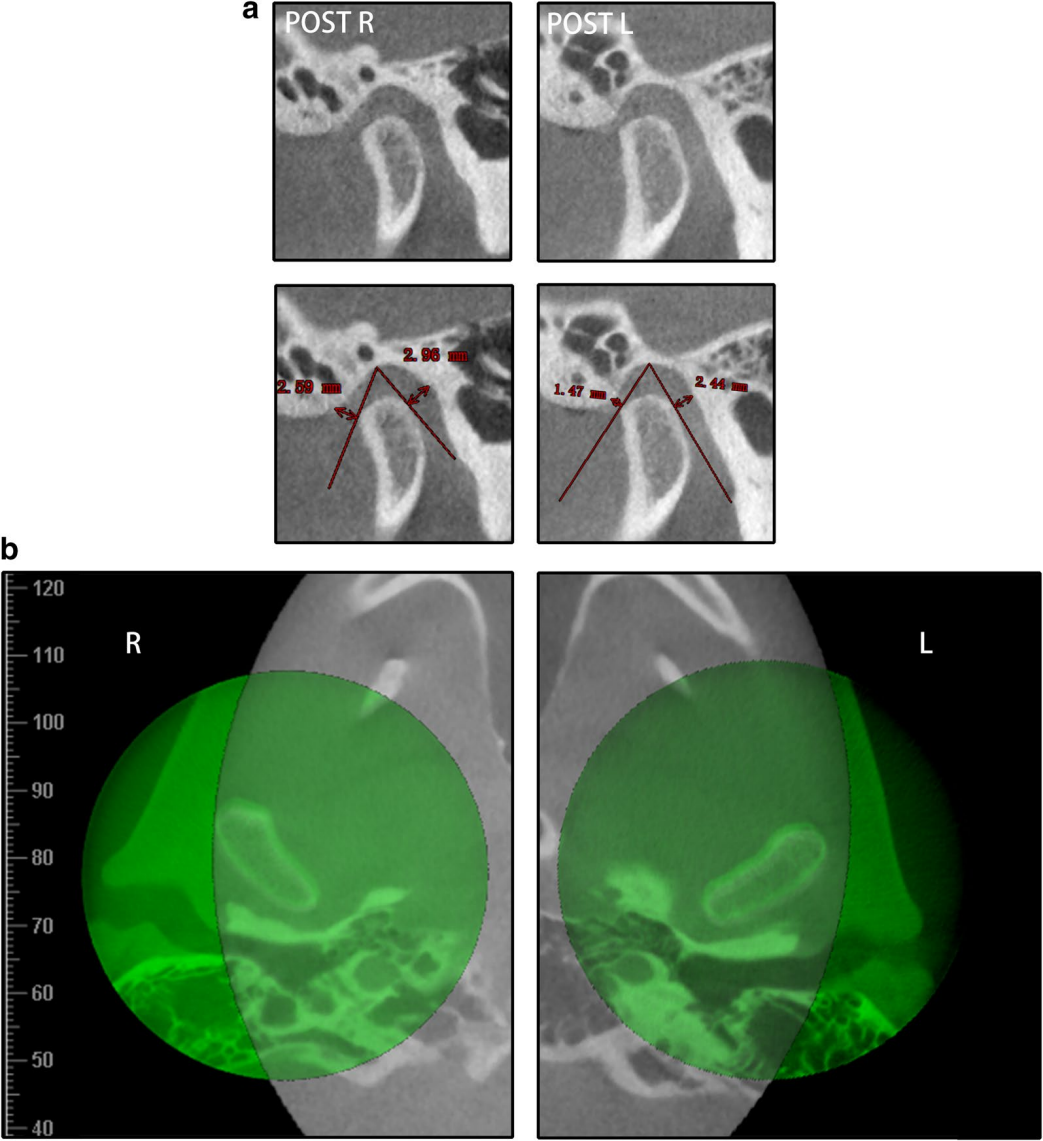
**a** The post-treatment bilateral TMJ JSIs. **b** CBCT superimposition of the pretreatment (gray) and post-treatment (green) bilateral TMJs

The revised Fig. 12 and legend is given below.
